# Localization and tissue tropism of the symbiont *Microsporidia MB* in the germ line and somatic tissues of *Anopheles arabiensis*

**DOI:** 10.1128/mbio.02192-23

**Published:** 2023-12-08

**Authors:** Edward E. Makhulu, Thomas O. Onchuru, Joseph Gichuhi, Fidel G. Otieno, Anne W. Wairimu, Joseph N. Muthoni, Lizette Koekemoer, Jeremy K. Herren

**Affiliations:** 1 International Centre of Insect Physiology and Ecology (ICIPE), Kasarani, Nairobi, Kenya; 2 Wits Research Institute for Malaria, University of the Witwatersrand, Witwatersrand, South Africa; 3 Department of Physical and Biological Sciences, Bomet University College, Bomet, Kenya; 4 Department of Biological Sciences, Brock University, St. Catharines, Ontario, Canada; University of Hawaii at Manoa, Honolulu, Hawaii; Uniwersytet Jagielloński w Krakowie, Cracow, Poland

**Keywords:** *Microsporidia MB*, malaria vectors, symbiotic microbes

## Abstract

The *Anopheles* symbiont, *Microsporidia MB*, is maternally inherited and has a strong malaria transmission-blocking phenotype in *Anopheles arabiensis. Microsporidia MB* is also vertically transmitted, sexually transmitted, and avirulent. These characteristics are expected to promote its spread through mosquito populations, enhancing the potential of *Microsporidia MB* as a candidate for the development of a symbiont-mediated malaria transmission-blocking strategy. We found that the patterns of *Microsporidia MB* localization over the development of *An. arabiensis* indicate accumulation in tissues linked to its transmission, specifically the male and female gonadal tissues. Transovarial vertical transmission of *Microsporidia MB* occurs in the female *An. arabiensis* ovary when *Microsporidia MB* becomes localized to the cytoplasm of the developing oocyte. In male *An. arabiensis*, *Microsporidia MB* is localized in the testis and vas deferens. Notably, a high intensity of *Microsporidia MB* can also be observed in the *An. arabiensis* adult but not larval gut. The levels of *Microsporidia MB* found in the female ovary are linked to the progression of oogenesis, increasing after blood feeding initiates the development of eggs. There was a significant change in *Microsporidia MB* levels in female and male *An. arabiensis* gonads, where intensity tended to decrease as mosquitoes aged. However, the intensities did not significantly change in the male or female guts. Altogether, the high specificity of *Microsporidia MB* tissue localization patterns and changes in infection prevalence and intensity suggest adaptation to maximize transmission and avirulence in *Anopheles arabiensis*.

**IMPORTANCE:**

*Microsporidia MB* is a symbiont with a strong malaria transmission-blocking phenotype in *Anopheles arabiensis*. It spreads in mosquito populations through mother-to-offspring and sexual transmission. The ability of *Microsporidia MB* to block *Plasmodium* transmission, together with its ability to spread within *Anopheles* populations and its avirulence to the host, makes it a very attractive candidate for developing a key strategy to stop malaria transmissions. Here, we report tissue tropism and localization patterns of *Microsporidia MB*, which are relevant to its transmission. We find that *Microsporidia MB* accumulates in *Anopheles arabiensis* tissues, linked to its sexual and vertical transmission. Its prevalence and intensity in the tissues over the mosquito life cycle suggest adaptation to maximize transmission and avirulence in *Anopheles arabiensis*. These findings provide the foundation for understanding the factors that may affect *Microsporidia MB* transmission efficiency. This will contribute to the development of strategies to maximize *Microsporidia MB* transmission to establish and sustain a high prevalence of the symbiont in *Anopheles* mosquito populations for malaria transmission blocking.

## INTRODUCTION

The malaria disease burden remains a major impediment to good health and economic development in many regions of sub-Saharan Africa. In 2021, a total of 247 million cases were reported, which resulted in 619,000 deaths, a strong indication that current control measures and their deployment levels are insufficient (1). Large-scale insecticide-treated net (ITN) distribution campaigns have had a significant impact on reducing the number of malaria cases (2). However, many malaria vectors have now developed resistance to the insecticides used in ITNs (3), and they are increasingly biting outdoors, where nets offer no protection (4). In addition, many malaria control efforts were significantly disrupted by the coronavirus disease 2019 pandemic (5), and the recent invasion of *Anopheles stephensi* across Africa is leading to a significant rise in malaria transmission (6–8). Altogether, these factors threaten to reverse the gains achieved for malaria reduction and indicate an urgent need for new strategies to control *Anopheles* mosquito populations or their capacity to transmit *Plasmodium* parasites.

A novel and potentially transformative method of controlling vector-borne disease involves the use of transmission-blocking symbionts. For dengue, a control strategy based on the transmission-blocking symbiont *Wolbachia* has been highly effective, and controlled field trials are currently implemented in over 13 countries (9). A similar strategy, based on a *Plasmodium* transmission-blocking symbiont in *Anopheles* mosquitoes, could be transformative for controlling malaria. The microsporidian symbiont, *Microsporidia MB*, is naturally found in anopheline mosquito populations and has been shown to block *Plasmodium* development (10). In addition, *Microsporidia MB* is both vertically and sexually transmitted (11). In conjunction with *Plasmodium* blocking, the high efficiency of vertical and sexual transmission of *Microsporidia MB*, which could facilitate the spread and maintenance of *Microsporidia MB* in *Anopheles* mosquito populations, has led to the suggestion that this symbiont could be deployed as a new tool for malaria control (12).

The success of a *Microsporidia MB*-based malaria control strategy will depend on the efficient vertical and horizontal transmission of the symbiont, which would enable the spread and maintenance of *Microsporidia MB* in *Anopheles* vector populations. For symbionts that are vertically and sexually transmitted, avirulence toward the host can be expected (13). In addition, vertically and sexually transmitted symbionts can be selected for their ability to enhance host fitness (14, 15). The tissue-level localization pattern and intensity of infection have been shown to play an important role in symbiont transmission and host fitness effects. In *Anopheles arabiensis*, *Microsporidia MB* is transmitted vertically from females to offspring and horizontally between males and females during mating, suggesting an important interaction with reproductive tissues (10, 11). Here, we investigated the tissue-level localization and changes in *Microsporidia MB* infection intensity across the development of *An. arabiensis*. We found that *Microsporidia MB* was predominately found in the reproductive organs of *An. arabiensis* males and females. Additionally, we observed *Microsporidia MB* inside developing oocytes in the *An. arabiensis* ovaries, indicating transovarial vertical transmission. Interestingly, the intensity of the *Microsporidia MB* infection in the female ovaries increased after blood feeding. The prevalence and intensity of *Microsporidia MB* infection in the female ovaries were found to decrease as *An. arabiensis* mosquitoes aged. In male *An. arabiensis*, *Microsporidia MB* is found in the testis and vas deferens, offering further confirmation of the basis of male-to-female sexual transmission as previously reported (11). *Microsporidia MB* is also found in the *An. arabiensis* adult gut at moderate prevalences. Notably, *Microsporidia MB* is always absent from the larval gut but is present in the larval body.

## RESULTS

### The *An. arabiensis* male and female gonads are the main sites of *Microsporidia MB* infection and localization in *An. arabiensis*

**FIG 1:**
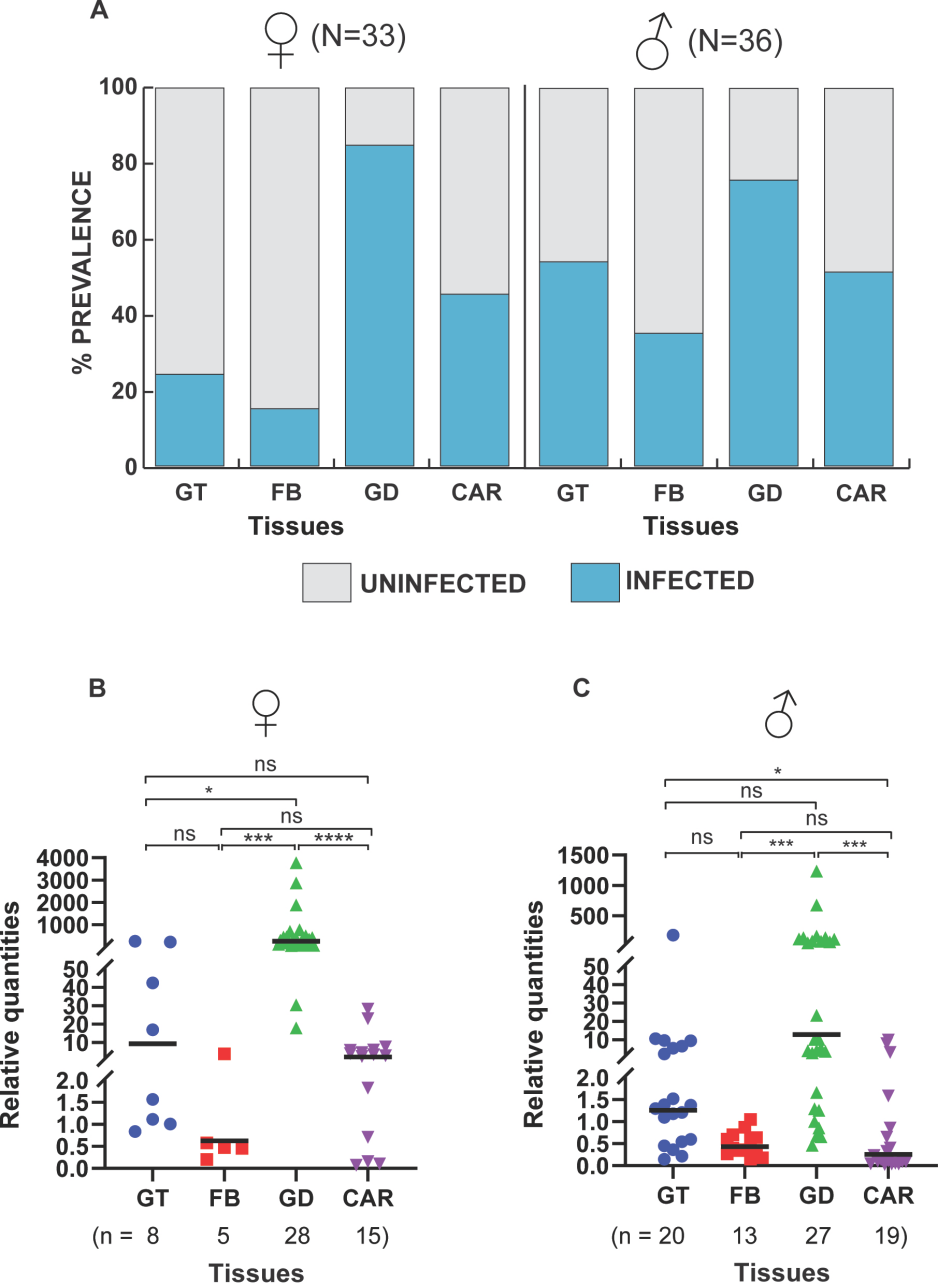
*Microsporidia MB* infection prevalence and intensity in adult *An. arabiensis* tissues. Prevalence rates and relative intensities of *Microsporidia MB* were measured for the guts (GT), fat bodies (FB), gonads (GD), and carcasses (CAR) of 7-day-old mosquitoes using qPCR. (**A**) The gonads were the tissues with the highest prevalence of *Microsporidia MB* in the dissected tissues, indicating that the gonads are the main site of infection. (**B and C**) Relative quantification of *Microsporidia MB* intensity in the tissues that turned out positive showed high infection intensities in the gonadal tissues in both females and males. The variations in the intensity of *Microsporidia MB* between the tissues were determined using the Kruskal-Wallis *H* test. A significant variation in the intensity was observed in both female tissues [*H*(3, *n* = 56) = 37.50, *P* < 0.0001] and male tissues [*H*(3, *n* = 79) = 36.70, *P* < 0.0001]. Post-hoc pairwise comparisons indicating the significance between the different groups were conducted and further affirmed gonads as the main sites of infection. Asterisks show the level of significance (*P  <  0.05, ***P  <  0.001, and ****P  <  0.0001), while ns indicates no significance. The bars in the plots indicate the median for each tissue group. The number of independent replicates is 7.

The prevalence and intensity of *Microsporidia MB* were investigated in the gonads, gut, fat body, and carcass of 7-day-old adult *An. arabiensis* by quantitative PCR (qPCR) (Fig. 1). We show that *An. arabiensis* male and female gonads had the highest *Microsporidia MB* prevalence, followed by the carcass, the gut, and the fat body (Fig. 1A). In females, *Microsporidia MB* infection prevalence was 24% in the guts, 15% in the fat body, 85% in the gonads, and 45% in the carcass, while for males, it was 56% in the guts, 36% in the fat body, 75% in the gonads, and 53% in the carcass. Notably, the prevalence in the guts of males was double that of female guts. Additionally, we measured and compared the relative intensity of *Microsporidia MB* (*Microsporidia MB* 18S RNA copies relative to the geometric mean of three host gene copy numbers) across all four tissues investigated (Table 1). We found that there was a significantly higher relative intensity of *Microsporidia MB* in the female gonads than in all other female tissues investigated (Fig. 1B). In males, the relative intensity of *Microsporidia MB* was significantly higher in the gonads than in the fat bodies and carcasses but not guts (Fig. 1C). In addition, the mean relative *Microsporidia MB* intensity in the male gut was significantly higher than that in the fat body and carcass, indicating that *Microsporidia MB* intensity can reach high levels in this tissue. In both sexes, the intensity of *Microsporidia MB* was lowest in the fat body (Fig. 1B and C). These findings indicate that the main site of *Microsporidia MB* infection in both male and female adult *An. arabiensis* is the gonadal tissue, and the gut and carcass are likely to be the minor sites of infection. This is in line with a study that investigated *Microsporidia MB* in only male *An. arabiensis* (11).

**TABLE 1:** Sequences of primers used for *Microsporidia MB* detection, *Anopheles* species identification, and amplification of reference genes for relative quantification of *Microsporidia MB*

Target	Primer name	Primer sequence (5′–3′)	Annealing temp.
*Microsporidia MB* 18S gene	MB18S 181F (10)	CGCCGGCCGTGAAAAATTTA	62°C
	MB18S 650R (10)	CCTTGGACGTGGGAGCTATC	
*An. gambiae* s.s. short interspersed elements (SINEs) 200	SINE 200F (16)	TCGCCTTAGACCTTGCGTTA	56°C
	SINE 200R (16)	CGCTTCAAGAATTCGAGATAC	
*Anopheles* ribosomal S7	S7UniF (10)	TCCTGGAGCTGGAGATGAAC	62°C
	S7UniR (10)	GACGGGTCTGTACCTTCTGG	
*An. gambiae s.s*. actin gene	Actin-F (this study)	AAGATGACGTGTTGTGCCCT	64°C
	Actin-R (this study)	ATTCCCGTTGCTGGAAGTGT	
*An. gambiae* s.s. GAPDH gene	An. ga. GAPDH-Aste-QPCR-F (this study)	GCGGTGGGCAAGGTCATCCC	64°C
	GAPDH-Aste-QPCR-R (17)	TTCATCGGTCCGTTGGCGGC	

### *Microsporidia MB* cells are identified by microscopy in oocytes in female and in the testis and gut in male *An. arabiensis*

**FIG 2:**
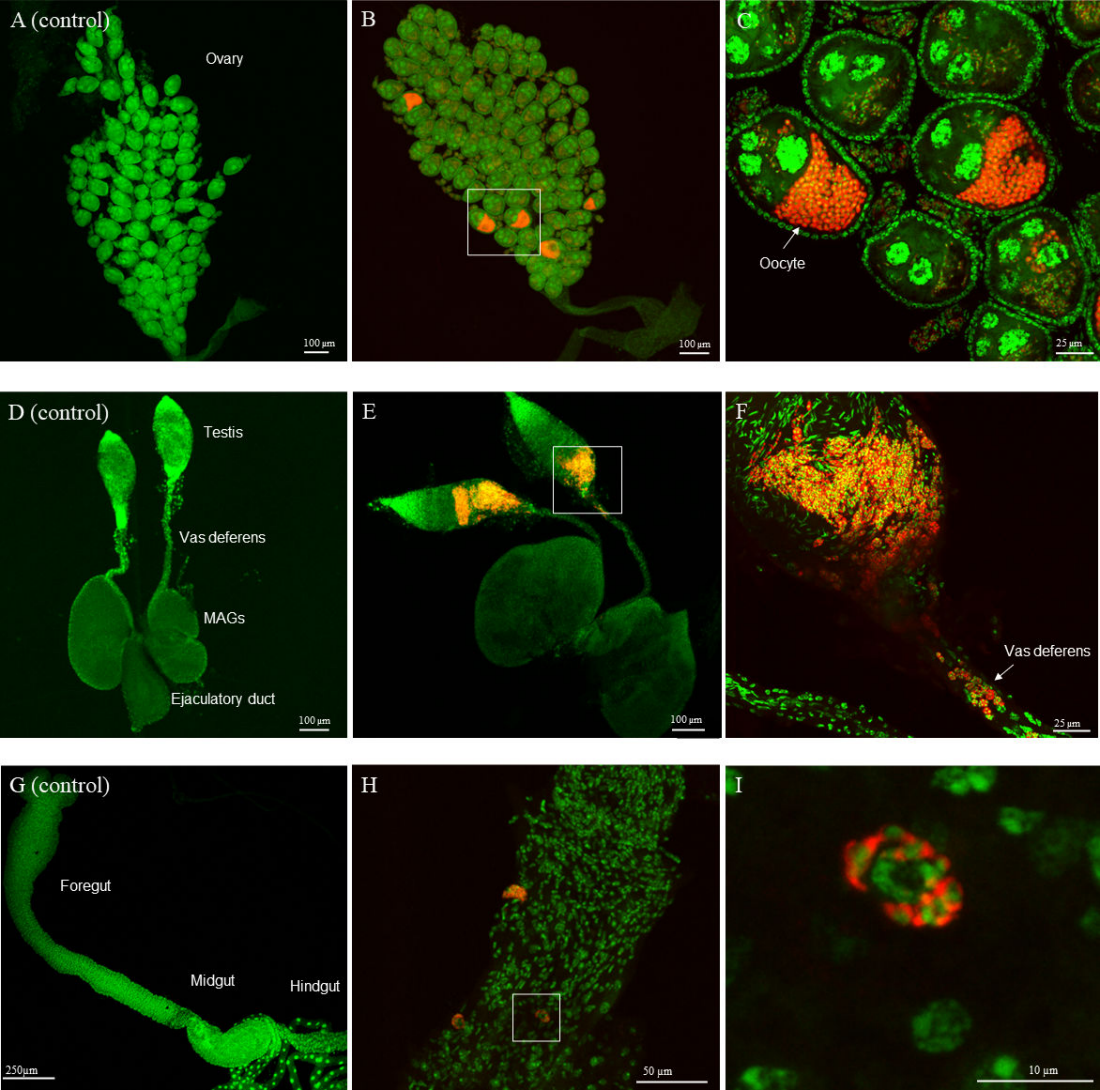
*Microsporidia MB* localization to cells in the male and female gonads and male gut. Localization of *Microsporidia MB* in the gonads and gut of 7-day-old *An. arabiensis* mosquitoes was conducted using FISH and confocal fluorescence microscopy. (**A**) Control *Microsporidia MB*-uninfected *An. arabiensis* female gonad. (**B**) *Microsporidia MB*-infected *An. arabiensis* female gonad. (**C**) A high intensity of *Microsporidia MB* is observed in vitellogenic oocytes. (**D**) Control *Microsporidia MB*-uninfected *An. arabiensis* male gonad. (**E**) *Microsporidia MB*-infected *An. arabiensis* male gonad. (**F**) A high intensity of *Microsporidia MB* is observed in parts of the testis and vas deferens that connect the testis to the ejaculatory duct. (G) Control *Microsporidia MB*-uninfected *An. arabiensis* male gut. (H) *Microsporidia MB*-infected *An. arabiensis* male gut (I) *Microsporidia MB* is observed in the *An. arabiensis* male midgut cells. The left panel in each row is a *Microsporidia MB* uninfected control tissue. The red signal in the images is the *Microsporidia MB* probe-CY5 FISH probe targeting *Microsporidia MB* while the green signal is the general DNA stain Sytox-Green. The number of independent replicates = 54 (ovaries), 11 (testis and vas deferens), and 7 (male guts).

To better understand where *Microsporidia MB* is located inside the male and female *An. arabiensis* gonads and guts, we visualized these tissues using fluorescent in situ hybridization (FISH) and confocal microscopy. The visualization of *Microsporidia MB* confirmed that male and female *An. arabiensis* gonads were the main sites of infection (Fig. 2A and B). In the *An. arabiensis* female gonad, *Microsporidia MB* is observed in all stages of egg chamber development (Fig. 2A). As the oocytes enter vitellogenesis, higher intensities of *Microsporidia MB* are observed. In the male gonad, *Microsporidia MB* is observed in the testis and vas deferens, in close proximity to developing sperm cells (Fig. 2B). In the *An. arabiensis* male gut, *Microsporidia MB* is occasionally observed at high infection levels in a small number of cells in the midgut and hindgut regions of males (Fig. 2C). We did not observe *Microsporidia MB* in the female guts, most likely because of the low prevalence rates in females and generally low-intensity infections in the guts.

### *Microsporidia MB* is not found in the larval gut and is higher in female adult mosquitoes

**FIG 3:**
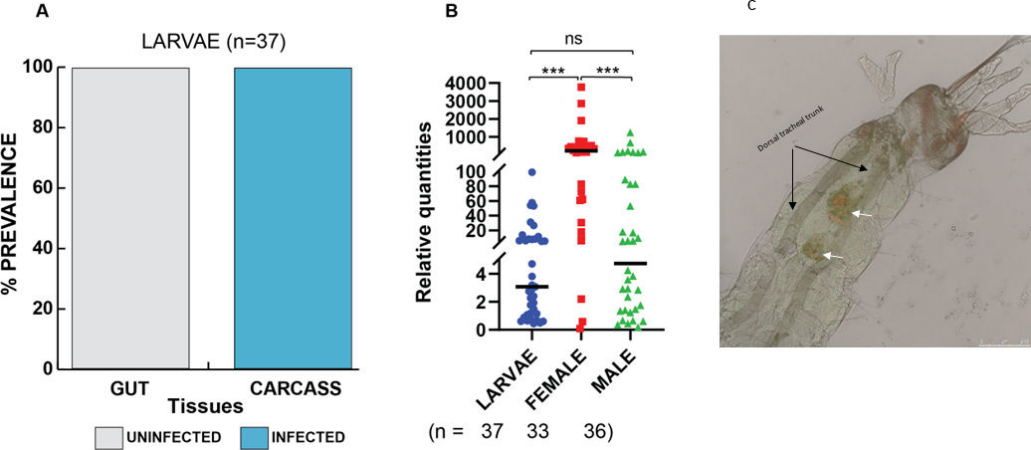
Localization of *Microsporidia MB* in larval tissues and comparison of its intensity between larvae and adult mosquito stages. L4 larvae and 1-week-old mosquitoes were used to compare *Microsporidia MB* infections in larvae and adult mosquitoes. (**A**) Using PCR, the presence and absence of *Microsporidia MB* were determined, and counts were used to calculate prevalence. Whereas there were no infected larval gut tissues, the larval carcass tissues had high prevalences. (**B**) A comparison of *Microsporidia MB* levels between the larvae, female, and male adult mosquitoes using qPCR revealed significant differences in the infection levels between these groups [*H*(3, *n* = 106) = 34.48, *P* < 0.0001]. Furthermore, post-hoc analysis with Dunn’s multiple comparisons test revealed that females have high levels of *Microsporidia MB*. The asterisks show the level of significance (***P  <  0.001), while ns represent no significance. The bars represent the median. The number of independent replicates is 13 (larvae) and 7 (adult mosquitoes). (**C**) Localization of *Microsporidia MB* in *An. arabiensis* larvae using FISH and confocal microscopy. FISH image of whole larvae merged with brightfield signal to show localization of *Microsporidia MB* (white arrows) in a distinct structure at the posterior end of the larvae. The red signal is the MB probe-CY5 FISH probe targeting *Microsporidia MB*, while the green signal is the general DNA stain Sytox-Green. The microscopy image shown is representative of five independent observations.

To better understand where *Microsporidia MB* is located during the larval stage of *An arabiensis*, L4 larval stages were dissected to separate the gut from the carcass, with carcasses containing all the remaining larval tissue, including the hemolymph. We observed that none of the gut samples were infected with *Microsporidia MB* (Fig. 3A). However, larval carcasses were found to have high *Microsporidia MB* infections, with a prevalence rate of 100% for all infected larvae investigated. The relative intensities of *Microsporidia MB* infection in *An. arabiensis* larvae were significantly less than those observed in adult female but not male mosquitoes (Fig. 3B). FISH and confocal microscopy analysis of the localization of *Microsporidia MB* in the larvae revealed that the symbiont is housed in a distinct structure that is located next to the gut epithelial cells (Fig. 3C).

### Blood meal affects the intensity of *Microsporidia MB* in the ovaries

**FIG 4:**
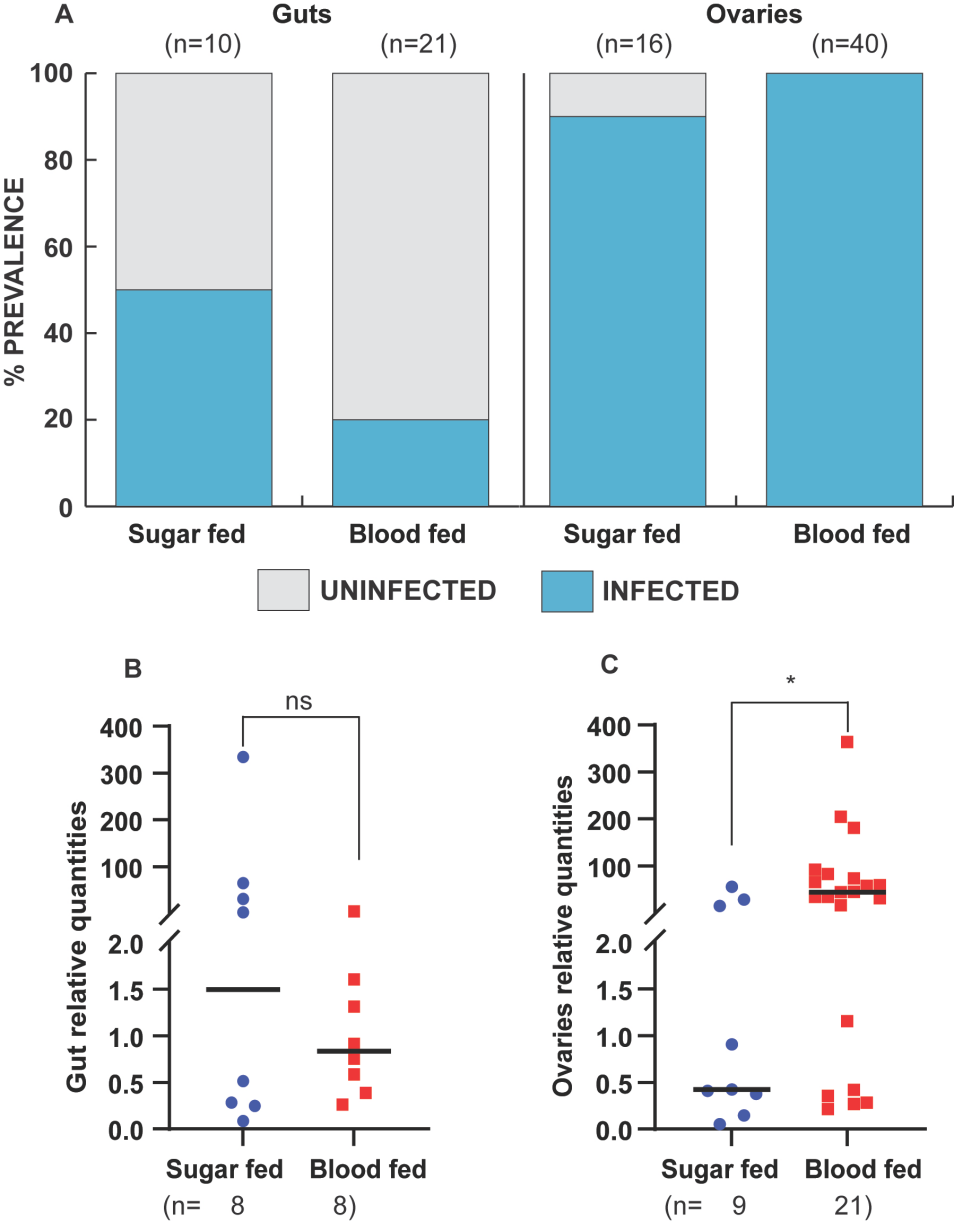
Effect of blood feeding on *Microsporidia MB* prevalence and intensity in female *An. arabiensis* gonads and gut tissues. Two- to three-day-old mosquitoes were fed on blood, and *Microsporidia MB* prevalence rates and infection levels were checked 5 days post-blood feeding and compared to sugar-fed controls using conventional PCR and qPCR, respectively. (**A**) The prevalence of *Microsporidia MB* in the guts and ovaries is expressed as a percentage of the tissues infected with *Microsporidia MB* compared to those uninfected. Blood feeding decreases prevalence in the gut but not in the gonads. (**B**) *Microsporidia MB* levels in the guts of the blood-fed group and the control group (fed on 10% glucose only) female mosquitoes were not different (two-tailed Mann–Whitney test, *P*  =  0.879, bars reflect the median). (**C**) In contrast to sugar-fed female mosquitoes, fed female mosquitoes have significantly higher intensities of *Microsporidia MB* in their ovaries (two-tailed Mann–Whitney test, *P*  =  0.019, bars reflect the median). The asterisk shows the level of significance (*P  <  0.05), while ns represents non-significance. The number of independent replicates is 4 (guts) and 2 (ovaries).

In *An. arabiensis*, oogenesis begins after female mosquitoes eclose but is arrested until females take a blood meal. To determine if the intensity of *Microsporidia MB* in *An. arabiensis* is affected by the onset of egg development and other physiological changes associated with taking a blood meal, we compared the prevalence and intensity of *Microsporidia MB* infection in the guts and gonads of *An. arabiensis* females that had fed on a blood meal with those that fed on sugar. In the *An. arabiensis* female gut, the prevalence of *Microsporidia MB* in sugar-fed mosquitoes was 53%, while for blood-fed mosquitoes, it was 20% (Fig. 4A). However, there was no change in the relative intensity of *Microsporidia MB* in the gut after blood feeding (Fig. 4B). The prevalence of *Microsporidia MB* infection was high in ovaries regardless of feeding status (Fig. 4A). However, we observed significantly higher relative intensities of *Microsporidia MB* in the gonads of blood-fed *An. arabiensis* than of sugar-fed controls (Fig. 4C).

### Age affects the intensity of *Microsporidia MB* in *An. arabiensis* gonads and guts

**FIG 5:**
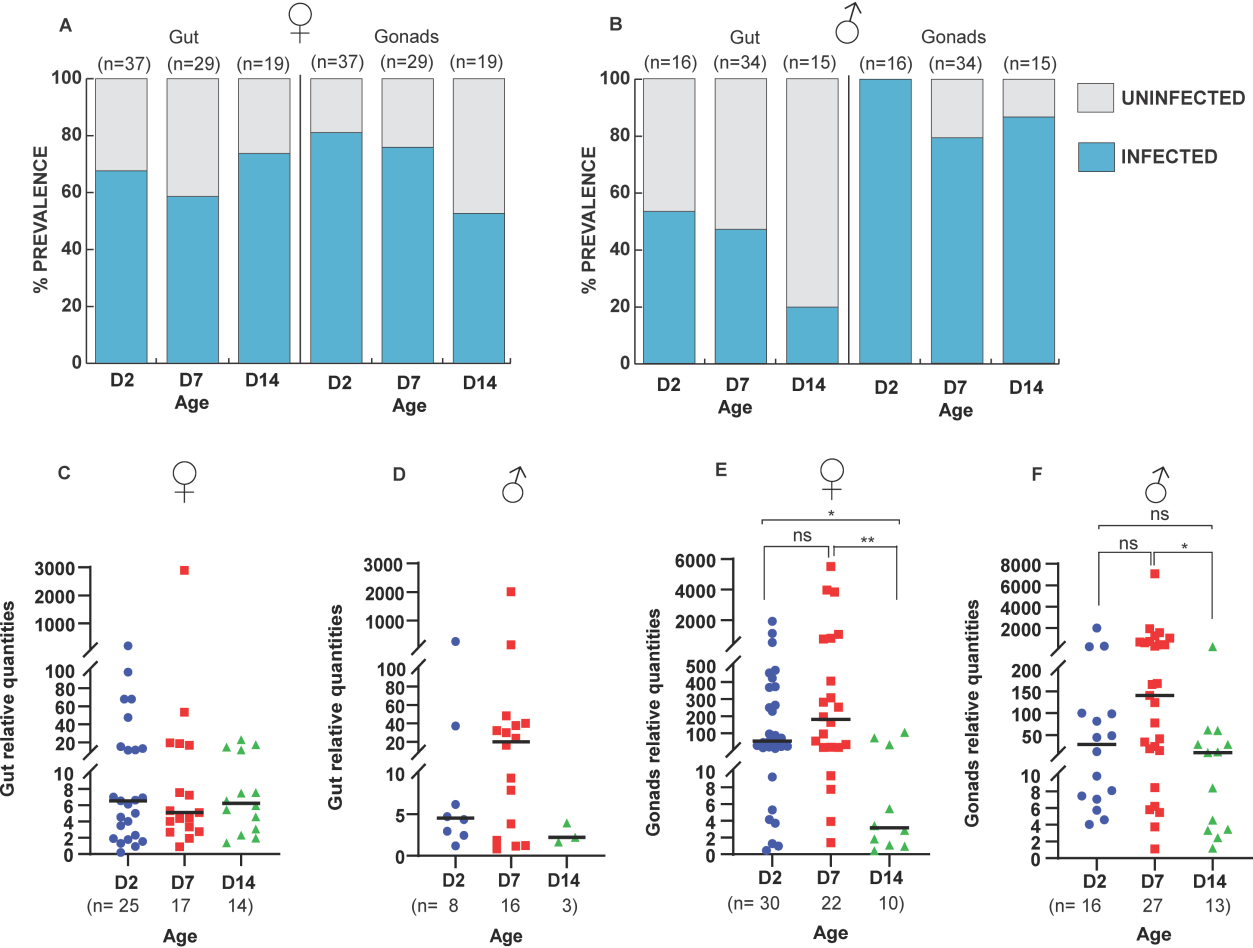
Effect of age on *Microsporidia MB* in *An. arabiensis* gonad and gut. The prevalence and intensity of *Microsporidia MB* infections were measured on 2-, 7-, and 14-day-old mosquitoes to determine how age affects *Microsporidia MB*. (**A and B**) In females, the prevalence of *Microsporidia MB* in the guts is higher at day 14 (74%) relative to day 2 (68%) and day 7 (59%), while in the gonads, the prevalence drops as mosquitoes age, with day 14 having the lowest (53%) compared to days 7 (76%) and 2 (81%). In males, the prevalence of *Microsporidia MB* in the guts drops as mosquitoes age over days 2 (53%), 7 (47%), and 14 (20%). However, the prevalence in the male gonads is relatively constant as the mosquitoes age (100%, 79%, and 87% for days 2, 7, and 14, respectively). (**C and D**) Intensities of *Microsporidia MB* in the guts of both female and male *An. arabiensis* were not affected by the age of the mosquitoes [female guts: *H*(2, *n* = 56) = 0.0098, *P* = 0.9951, male guts: *H*(2, *n* = 27) = 2.602, *P* = 0.2723]. (**E**) In the female gonads, age significantly affected the relative intensities of *Microsporidia MB* [*H*(2, *n* = 62) = 10.96, *P* = 0.0042]. Upon conducting post-hoc analysis with Dunn’s multiple comparisons test, there was a significant decrease at day 14 compared to days 2 and 7. (**F**) Similarly, the relative intensities in the male gonads were significantly affected by mosquito age [*H*(2, *n* = 56) = 8.782, *P* = 0.0124]. Upon conducting post-hoc analysis with Dunn’s multiple comparisons test, there was a significant decrease only at day 14 compared to day 7. Asterisks show the level of significance (*P  <  0.05, **P  <  0.01), while ns indicates no significance. The number of independent replicates is 19.

To investigate the effect of aging on the prevalence and intensity of *Microsporidia MB* in *An. arabiensis* gonadal and gut tissue, male and female *An. arabiensis* were aged for 2, 7, and 14 days prior to dissection of tissues and quantification of *Microsporidia MB* by qPCR. The prevalence of *Microsporidia MB* in the guts of females remained relatively stable, while the prevalence in the gonads had a decreasing trend as mosquitoes aged (Fig. 5A). The prevalence of *Microsporidia MB* in the guts of male *An. arabiensis* decreased as mosquitoes aged, with 14-day-old males having the lowest infection rate (Fig. 5B). Similarly, the prevalence of male gonads decreased with age (Fig. 5B). In both male and female guts, there was no significant change in *Microsporidia MB* intensity across the different age groups (Fig. 5C and D). There was a significant change in the relative intensity of *Microsporidia MB* in female and male *An. arabiensis* gonads, where intensity tended to decrease as mosquitoes aged (Fig. 5E and F). Pairwise comparisons post-hoc analysis with Dunn’s multiple comparisons test revealed a significant decrease between days 2 and 14 (*P* = 0.032) and days 7 and 14 (*P* = 0.003) in females (Fig. 5E), while in males, a significant decrease was only observed between days 7 and 14 (*P* = 0.011) (Fig. 5F). These results suggest that age affects *Microsporidia MB* infection levels only in the gonads but not the guts in both males and females.

## DISCUSSION

Our findings demonstrate that the gonads of male and female *An. arabiensis* are the main sites of *Microsporidia MB* infection. This suggests that maternal and sexual transmission are likely to be the most important *Microsporidia MB* infection routes. Both maternal and sexual transmission routes require that *An. arabiensis* hosts do not incur significant fitness costs when infected with *Microsporidia MB* (13). In line with this, *Microsporidia MB* does not have any known major effect on key metrics of fitness, including larval developmental time and pupation rate, fecundity of female mosquitoes, and survival rate of infected adult mosquitoes (10).

Other insect endosymbionts, including *Wolbachia*, have intensity-dependent effects on host fitness (18). It is therefore possible that very high-intensity *Microsporidia MB* infections do have a fitness cost, but this would need further investigation. The link between *Wolbachia* intensity and host fitness is complex, and, in many cases, fitness is not affected by high endosymbiont intensities (19). It is likely that the pattern of endosymbiont localization in host tissues has important fitness consequences for endosymbionts such as *Wolbachia* and *Microsporidia MB*. A strain of *Wolbachia*, *w*MelPop, that overproliferates and decreases host fitness has been found at high intensities in the central nervous system and muscles of its insect hosts (20, 21). It is possible that endosymbionts mitigate effects on host fitness by limiting most of the infection and proliferation to certain tissues.

Our findings indicate that *Microsporidia MB* is primarily found in gonadal tissue, and this is a suggestive adaptation to minimize host fitness costs without compromising transmission rates. Fluorescence confocal microscopy revealed that *Microsporidia MB* was present in oocytes and nurse cells across all stages of oogenesis. These findings clearly demonstrate that *Microsporidia MB* maternal transmission is transovarial, which explains the need for higher *Microsporidia MB* intensities to be maintained in the female gonads.

In the male germline, *Microsporidia MB* is primarily localized to the testis and vas deferens. The testis is the site of sperm production, comprising a proximal end with stem cell divisions, spermatocysts in different stages of development, and a distal spermatozoa reservoir (22). In *An. arabiensis*, we observed *Microsporidia MB* cells as clusters in the spermatocyst and spermatozoa reservoir regions of the testis and, more rarely, in the stem cell region. These findings suggest that *Microsporidia MB* is packaged with spermatozoa at their early development stages.

We observed very low levels of *Microsporidia MB* in the *An. arabiensis* adult fat body. Pathogenic Microsporidians are known to infect and proliferate in the insect’s fat body (22). It is likely that, as the primary energy storage tissue, fat bodies provide the nutrients required for this proliferation. The low levels of *Microsporidia MB* in the adult fat body of *An. arabiensis* could be part of the explanation for its avirulence in *An. arabiensis*. The moderate levels of *Microsporidia MB* in the carcass samples, which contained all the tissues apart from the gut, gonads, and fat body, suggest that we cannot exclude there being another *Microsporidia MB* infection reservoir in adult *An. arabiensis*.

In the adult *An. arabiensis* gut, there were a small proportion of samples that had very high intensities of *Microsporidia MB.* The significance of these high-intensity gut infections is yet to be resolved. Fluorescence confocal microscopy revealed that *Microsporidia MB* was found at very high intensities in a small subset of cells in the adult male mid and hindgut. The shape and positioning of these cells suggest that they could be progenitor cells (intestinal stem cells and enteroblasts) (23). Progenitor cells are important for cell replenishment in the gut in response to intrinsic factors that bring about gut epithelium turnover (23). It has been shown that there are low levels of progenitor cells in *Anopheles gambiae* mosquitoes (24). This could explain the overall low prevalence in the gut tissues in the various experiments.

It is notable that, in contrast to adults, *Microsporidia MB* was never observed in the larval gut. A possible explanation for this is that its presence in the larval gut could have prohibitive fitness costs since larvae need to develop rapidly and do so by feeding continuously (25). Furthermore, larval guts are made up of regenerative endoreplicating cells that account for a greater portion of the midgut epithelial cells. This differs from adult guts, where midgut epithelial cells are derived from progenitor cells (26, 27). The absence of *Microsporidia MB* in the gut contradicts our previous report on its presence in the larval epithelial tissues (10). In the current study, we have conducted exhaustive additional FISH and confocal microscopy experiments as well as qPCR (which was not used in the original experiments). Based on this new data, we are no longer able to observe *Microsporidia MB* in the larval gut. We do, however, find that *Microsporidia MB* is localized in a distinct structure that is proximal to the larval gut, which likely explains our previous characterization. The high *Microsporidia MB* prevalence rates in the larval carcass suggest that *Microsporidia MB* establishes itself in the host tissues at a very early stage of *An. arabiensis* development cycle. The higher overall intensity of *Microsporidia MB* in adult females compared to adult males and larvae suggests a higher replication rate of the symbiont in females and may suggest that vertical transmission is the primary mode of transmission. We noted variations in prevalence rates and intensities in the F1s used across experiments. This is likely due to variations in transmission rates and seasonal and environmental influences on *Microsporidia MB* prevalences in the field-collected mothers (10).

We also investigated how localization and *Microsporidia MB* intensity change as mosquitoes age and upon blood feeding. In *An. arabiensis*, oogenesis starts when mosquitoes eclose but is then paused until females take a blood meal (28). Once females take a blood meal, several physiological changes occur that enable the blood to be digested and ultimately meet the nutritional demands of egg production (29). Since there are major changes occurring in the gut and gonads of females post-blood feeding, we investigated the changes in the levels of *Microsporidia MB* in these tissues.

We observed a moderate decrease in the prevalence of *Microsporidia MB* in female *An. arabiensis* guts after blood feeding. However, the levels of *Microsporidia MB* in the gut did not decrease significantly in response to blood feeding. It is possible that the decrease in prevalence could be linked to the process of gut epithelium renewal, which occurs after blood feeding (23). In contrast, we observed a marked increase in the intensity of *Microsporidia MB* in the female gonad after blood feeding. Notably, *Microsporidia MB* is also observed by microscopic examination at high intensities in the later (vitellogenic) stages of oogenic development, which develop after the blood meal-induced initiation of oogenesis (26). Therefore, the proliferation of *Microsporidia MB* in vitellogenic oocytes is a possible explanation for the increase in the intensity of *Microsporidia MB* after blood feeding.

We did not observe *Microsporidia MB* levels increasing in male or female guts and gonads as *An. arabiensis* mosquitoes aged. This pattern of growth is consistent with symbionts that regulate proliferation to mitigate fitness costs (28). Notably, there was a significant decrease in the prevalence and intensity of *Microsporidia MB* in female gonads between 7 and 14 days of aging. This may indicate a loss of *Microsporidia MB* from germline cells after numerous rounds of division and an inability for these cells to be re-infected. However, the aged *An. arabiensis* female mosquitoes had not been blood-fed and therefore had not completed any gonotrophic cycles, which could be expected to lower the rate of stem cell division (29). In addition, we observed a decrease in the prevalence and intensity of *Microsporidia MB* in male gonads between 7 and 14 days of aging. This could be linked to the loss of *Microsporidia MB* from stem cells after multiple rounds of division and the shedding of *Microsporidia MB* in the male ejaculate, or perhaps it reflects that *Microsporidia MB* can only survive for a limited time in the male ejaculatory duct.

Overall, our findings suggest that *Microsporidia MB* localization patterns may serve to maximize transmission success while minimizing negative effects on host fitness. *Microsporidia MB* has proliferation and localization patterns that are consistent with a co-evolved symbiont that exploits sexual and vertical transmission routes. A better understanding of changes in localization and symbiont intensity across the *An. arabiensis* lifecycle will contribute to improving *Microsporidia MB*-infected mosquito rearing and aid in the formulation of strategies to disseminate *Microsporidia MB*.

## MATERIALS AND METHODS

### Mosquito sample collection

Wild-caught gravid adult female *Anopheles gambiae s.l.* were collected using mouth aspiration. Collected mosquitoes were identified morphologically to the species level, and sub-species identification of *Anopheles gambiae s.l.* was carried out by PCR (16). Sampling was conducted from one site in Kenya, the Ahero irrigation scheme (−34.9190W, −0.1661N). The mosquitoes used in this study were collected between September 2021 and October 2021. Sampled mosquitoes were transported to the ICIPE—Duduville campus in Nairobi and maintained on 10% glucose and water.

### Mosquito processing and development of isofemale lines

Wild collected mosquitoes were maintained in an insectary at 27 ± 2.5°C, humidity 60%–80%, and 12-h day and 12-h night cycles and induced to oviposit in individual microcentrifuge tubes containing a wet 1 cm × 1 cm Whatman filter paper. Eggs from each female were counted under a compound microscope using a paintbrush and then dispensed into water troughs for larval development at 30.5°C and 30%–40% humidity. Tetramin baby fish food was used to feed developing larvae. Upon laying eggs, the G_0_ females were screened for the presence of *Microsporidia MB* by PCR. The larval offspring of *Microsporidia MB*-positive field-caught female mosquitoes were transferred into larval rearing troughs for further development. Upon reaching the L4 larval stage, representative samples from each positive isofemale line were screened to determine the *Microsporidia MB* infection status of the line. Mosquitoes were maintained as adults; this was done in an insectary at 30°C with a relative humidity of 75%, 12-h day and 12-h night cycles, and a feeder with a 10% glucose solution.

### Larval and adult dissection

*An. arabiensis* G1 larvae and adult mosquitoes were dissected using forceps under the Zeiss Stemi 2000-C stereomicroscope to obtain G1 L4 larval and adult *Anopheles* tissues. During the dissection of the L4 larvae, alive samples were placed on a drop of 1× phosphate buffered saline (PBS). The larvae were restrained at the junction between the head and thorax with a pair of forceps. A dissecting needle pin was then used to probe the intersection between the second and last abdomen segments. The siphon and saddle of the larvae were held and pulled gently to obtain the gut of the larvae using a new pair of forceps. The carcass and the gut were placed in separate 1.5 microcentrifuge tubes containing 20 µL of 1× PBS and 0.5 mm zirconium beads for DNA extractions. For adults, G1 mosquito samples were first anesthetized for a few minutes until immobilized by aspirating them into 1.5 microcentrifuge tubes and placing them in ice. Upon immobilization, the mosquitoes were placed in a drop of 1× PBS on a glass slide. The junction between the last and second-last abdomens was probed using a dissecting needle. The last segment was gently pulled to release the gonads and the gut. The gut and gonads were separated and placed in separate 1.5 microcentrifuge tubes containing 20 µL of 1× PBS and 0.5 mm zirconium beads ready for nucleic acid extractions. Similarly, the fat bodies and carcasses (all remaining tissue) were also separated and placed in separate 1.5 microcentrifuge tubes, as described above. Forceps were sterilized after every dissection to prevent contamination.

Individual adult mosquitoes and larvae used in all experiments were pre-screened, and those with at least one tissue(s) positive for *Microsporidia MB* were then considered the final sample sizes for prevalence and intensity determination in all the data generated. The prevalence of *Microsporidia MB* in the dissected tissues was obtained by counting tissue samples that turned out positive after PCR screening and expressed as a percentage of the final sample size. To compare the intensities of *Microsporidia MB* between mosquito adults and larvae, summations of the relative intensities from the dissected adult tissues (used in tissue localization experiments) and those from larvae carcasses were compared.

### Mosquito blood feeding

Two- to three-day-old *Microsporidia MB*-positive and -negative *An. arabiensis* mosquitoes maintained at a temperature of 30°C and a relative humidity of 75% were starved for 2 h without water or glucose in preparation for blood feeding. Membrane feeding was conducted according to reference (30). Briefly, bovine blood was transferred into a Hemotek feeding apparatus (Hemotek, UK) with a Parafilm-A membrane set at 37°C. The Hemotek feeding apparatus was placed on top of a cage of starved *An. arabiensis* mosquitoes, and feeding was allowed to take place for 1 h. Fully engorged *An. arabiensis* were transferred to new cages, maintained for 5 days post-blood feeding, and later dissected to obtain the gut and ovaries, which were screened for *Microsporidia MB* presence and levels.

### Flourescent *in situ* hybridisation localization

Dissected tissues were fixed overnight in 4% paraformaldehyde at 4°C. After fixation, the samples were rehydrated in 50% ethanol for 30 minutes and transferred to 1× PBS for another 30 minutes. FISH was then conducted to localize *Microsporia MB* within the tissues. Hybridization was done by incubating the tissues in 100 µL of hybridization buffer overnight at 50°C. The FISH hybridization mix contained hybridization buffer (dH_2_O, 5 M NaCl, 1 M Tris/HCl [pH = 8, and 10% SDS), a *Microsporidia MB*-specific CY5 probe (10), a 0.5 µM final concentration, and SYTOX Green general DNA staining. After staining, the samples were washed twice with 100 µL of prewarmed wash buffer (dH_2_O, 5 M NaCl, 1 M Tris/HCl [pH = 8], 0.5 M EDTA, and 10% SDS). The tissues were then placed on a slide and visualized immediately using a Leica SP5 confocal microscope (Leica Microsystems, USA). The images were analyzed with the ImageJ 1.50i software package (31).

### DNA extraction and molecular detection and quantification of *Microsporidia MB*

DNA was extracted using the ammonium acetate protein precipitation method (10). Extracted DNA samples were screened to determine their *Microsporidia MB* infection status using the *Microsporidia MB*-specific primers (Table 1) (10). The PCR reaction used in detection comprised a 10 µL reaction consisting of 2 µL HOTFirepol Blend Master mix Ready-To-Load (Solis Biodyne, Estonia), 0.5 µL of 5 pmol/µL forward and reverse primers, 2 µL of the DNA template, and 5 µL nuclease-free PCR water. The thermocycling conditions employed were initial denaturation at 95°C for 15 min, followed by 35 cycles of denaturation at 95°C for 1 min, annealing at 62°C for 90 s, and extension at 72°C for a further 60 s. Final elongation was done at 72°C for 5 min. Samples positive for *Microsporidia MB* were subjected to relative qPCR analysis to quantify infection levels and species ID (10, 16). The qPCR analysis involved the MB18SF and MB18SR primers, normalized with the geometric means of cycle threshold values for the primers targeting the *Anopheles* ribosomal S7 gene, Actin gene, and GAPDH gene (Table 1) as the reference host genes. The 10 µL PCR reaction consisted of 2 µL HOT FIREPol EvaGreen HRM no ROX Mix Solis qPCR Master mix (Solis Biodyne, Estonia), 0.5 µL of 5 pmol/µL forward and reverse primers, 2 µL of the DNA template from *Microsporidia MB*-positive samples, and 5 µL nuclease-free PCR. The thermocycling conditions employed were an initial denaturation at 95°C for 15 min, followed by 35 cycles of denaturation at 95°C for 1 min, annealing at 62°C for 90 s, and extension at 72°C for a further 60 s. Finally, melting curves were generated by melting analysis with temperature ranges from 65°C to 95°C. The PCR and qPCR were carried out on a MIC qPCR cycler (BioMolecular Systems, Australia). Each sample was confirmed to have the characteristic melt curve associated with the *Microsporidia MB* MB18SF/MB18SR primers.

### Data analysis

All the data were tested for normality using the Shapiro-Wilk test. For non-normal unpaired data, a two-tailed Mann–Whitney *U* test was used to determine the difference between the groups. In cases with more than two data groups, we used the Kruskal-Wallis H test to determine the significance of differences between the groups. If significant, we carried out Dunn’s post-hoc test. All statistical analyses were performed using Graphpad Prism version 6.0 c software or R (version 8.0.2). *P*-values of *P  <  0.05, **P  <  0.01, ***P  <  0.001, and ****P  <  0.0001 were deemed to be statistically significant. The percentage of tissues with *Microsporidia MB* infection compared to those without infection was used to express the prevalence data using Adobe Illustrator (AI) (version 23.0.1). Figures were created and/or edited using AI.

## Data Availability

The PCR and qPCR data are available in the Dryad Digital Repository at https://doi.org/10.6084/m9.figshare.24637809.v1.

## AFTER EPUB

*[This article was published on 8 December 2023 with missing material in Acknowledgments. The Acknowledgments were corrected in the current version, posted on 14 December 2023.]*
